# Diagnostic Accuracy of Point-of-Care Ultrasound for Intussusception in Children Presenting to the Emergency Department: A Systematic Review and Meta-analysis

**DOI:** 10.5811/westjem.2020.4.46241

**Published:** 2020-07-02

**Authors:** Margaret Lin-Martore, Aaron E. Kornblith, Michael A. Kohn, Michael Gottlieb

**Affiliations:** * University of California, San Francisco, Department of Emergency Medicine and Pediatrics, San Francisco, California; † Stanford University, Department of Emergency Medicine, Stanford, California; ‡ University of California, San Francisco, Department of Epidemiology & Biostatistics, San Francisco, California; § Rush University Medical Center, Department of Emergency Medicine, Chicago, Illinois

## Abstract

**Introduction:**

Ileocolic intussusception is a common cause of pediatric bowel obstruction in young children but can be difficult to diagnose clinically due to vague abdominal complaints. If left untreated, it may cause significant morbidity. Point-of-care ultrasound (POCUS) is a rapid, bedside method of assessment that may potentially aid in the diagnosis of intussusception. The purpose of this systematic review and meta-analysis was to determine the diagnostic accuracy of POCUS for children with suspected ileocolic intussusception by emergency physicians (EP).

**Methods:**

We conducted a systematic search on PubMed, Embase, CINAHL, LILACS, the Cochrane databases, Google Scholar, as well as conference abstracts, and assessed bibliographies of selected articles for all studies evaluating the accuracy of POCUS for the diagnosis of intussusception in children. We dual extracted data into a predefined worksheet and performed quality analysis with the QUADAS-2 tool. Data were summarized and a meta-analysis was performed.

**Results:**

Six studies (n = 1303 children) met our inclusion criteria. Overall, 11.9% of children had intussusception. POCUS was 94.9% (95% confidence interval [CI], 89.9% to 97.5%) sensitive and 99.1% (95% CI, 94.7% to 99.8%) specific with a likelihood ratio (LR)+ of 105 (95% CI, 18 to 625) and a LR− of 0.05 (95% CI, 0.03 to 0.10).

**Conclusion:**

POCUS by EPs is highly sensitive and specific for the identification of intussusception for children presenting to the emergency department.

## INTRODUCTION

Ileocolic intussusception is the most common cause of gastrointestinal obstruction in children and represents a common abdominal emergency in early childhood.^1^ As the ileum telescopes into the cecum, the mesentery is compressed, which leads to venous and lymphatic bowel congestion. As time passes, the process can lead to ischemia, perforation, peritonitis, and significant morbidity. Therefore, rapid diagnosis is paramount. Children with intussusception may present with nonspecific symptoms such as vomiting, abdominal pain, or lethargy.^1^ The classic triad of colicky abdominal pain, palpable abdominal mass, and bloody stool are present in less than 50% of children with intussusception, which can make the diagnosis challenging to make on history and physical examination alone.^2^ Additionally, since the majority of cases are seen in children aged 6–36 months,^1^ the history is often limited, which can compound the difficulty of diagnosis.

Ultrasound is considered the first-line diagnostic test of choice when evaluating children for intussusception because of its high accuracy and lack of harmful ionizing radiation.^3^ Radiology-performed ultrasound has been shown to have excellent test characteristics, with high sensitivity (98%) and specificity (98%),^4^ and is far superior to abdominal plain radiography in accurately evaluating children for intussusception.^5^ Moreover, ultrasound for the evaluation of ileocolic intussusception is relatively uncomplicated to learn and can be accurately performed by junior radiology trainees.^6^ Still, radiology-performed ultrasound requires a capable provider, often including a technician and/or radiologist. Such expertise may not be available 24 hours a day at many institutions. Delays from limited access to radiology-performed ultrasound may lead to increased morbidity and mortality.^7^

Point-of-care ultrasound (POCUS) is increasingly used in adult and pediatric emergency medicine for a wide range of applications.^8^–^10^ POCUS for the evaluation of ileocolic intussusception may allow EPs to make the diagnosis at the patient’s bedside and avoid delays in diagnosis. However, it is important to determine the diagnostic accuracy of this approach prior to routine use. The purpose of this systematic review and meta-analysis was to determine the diagnostic accuracy of POCUS in children with possible intussusception by EPs.

## METHODS

Our study conforms to the Preferred Reporting Items for Systematic Reviews and Meta-analyses guidelines and was conducted in accordance with best practice recommendations.^11^ The study was also registered with PROSPERO, the international prospective register of systematic reviews (CRD42019122126).

We conducted a systematic search of PubMed, Embase, the Cumulative Index of Nursing and Allied Health (CINAHL), Latin American and Caribbean Health Sciences Literature database (LILACS), Google Scholar, the Cochrane Database of Systematic Reviews, and the Cochrane Central Register of Controlled Trials to include citations from inception through January 14, 2019. A medical librarian assisted us in our search. In accordance with the recommendations by Bramer and colleagues, only the top 200 Google Scholar search terms were selected.^12^ Details of our search strategy are included in the Appendix. In addition to the above, we also hand searched the last five years of conference abstracts from the American College of Emergency Physicians and the Society for Academic Emergency Medicine and the last three years of abstracts from the American Institute of Ultrasound in Medicine (only three years were available) for relevant abstracts. We also reviewed the references of identified studies and review articles for potentially missed articles.

Inclusion criteria consisted of all prospective or retrospective studies assessing the accuracy of POCUS for intussusception in pediatric patients (defined as younger than 18 years of age). There were no language or date restrictions. All studies had to include a gold standard confirmatory test (ie, radiology-performed ultrasound, other radiology imaging, air enema, or patient follow-up). We excluded case reports, case series, studies on practice patients, and adult studies.

Two investigators (MLM, AEK) independently assessed studies for eligibility based on the above criteria. All abstracts meeting inclusion criteria underwent full-text review. Studies determined to meet criteria after full-text review by both investigators were included in the final data analysis. Discrepancies were resolved by consensus between the two investigators and a third party (MG). Two investigators (MLM, AEK) independently extracted data from the included studies. The investigators were trained on extraction and used a predesigned data collection form. The following information was extracted: first author; year; study design; type of publication (ie, abstract or full article); sample size; country; study location (ie, pediatric emergency department [PED], other); median/mean age of patients; number of male patients; ultrasonographer training level (ie, trainee, attending); ultrasound training protocol; ultrasound probe and machine; scanning protocol; gold standard; intussusception rate; true-positive results; false-positive results; true-negative results; and false-negative results. Studies were independently assessed for quality by two investigators (MLM, AEK) using the Quality Assessment of Diagnostic Accuracy Studies–2 (QUADAS-2) Tool.^13^ Discrepancies were resolved by consensus between the two investigators and a third party (MG).

The results were pooled from the included studies using a bivariate mixed-effects model to calculate sensitivity, specificity, positive likelihood ratio (LR+), and negative likelihood ratio (LR−) with 95% confidence intervals (CI).^14^ We constructed a summary receiver operating characteristic (sROC) curve with observed study data, and calculated the area under the curve. We assessed heterogeneity between studies graphically by plotting their sensitivity/specificity points on the sROC grid, creating standard forest plots of sensitivity and specificity, and calculating *I*^2^.^15^ We also performed a sensitivity analysis after excluding one study^16^ that appeared to be an outlier due to physician training, index test, and reference test. We performed additional sensitivity analyses excluding retrospective studies as well as excluding studies that were reported as abstracts only. We assessed the possibility of publication bias using a scatter plot of the inverse of the square root of the effective sample size vs the diagnostic log odds ratio and reported the p-value for Deeks’ funnel plot asymmetry test.^17^ Statistical analysis was completed with Stata/SE, version 15.1 (StataCorp LP, College Station, TX). We used the MIDAS module to perform analyses and construct the figures. For subgroups of fewer than four studies, we used MetaDTA (https://crsu.shinyapps.io/dta_ma/) to pool results.

## RESULTS

**Figure 1:**
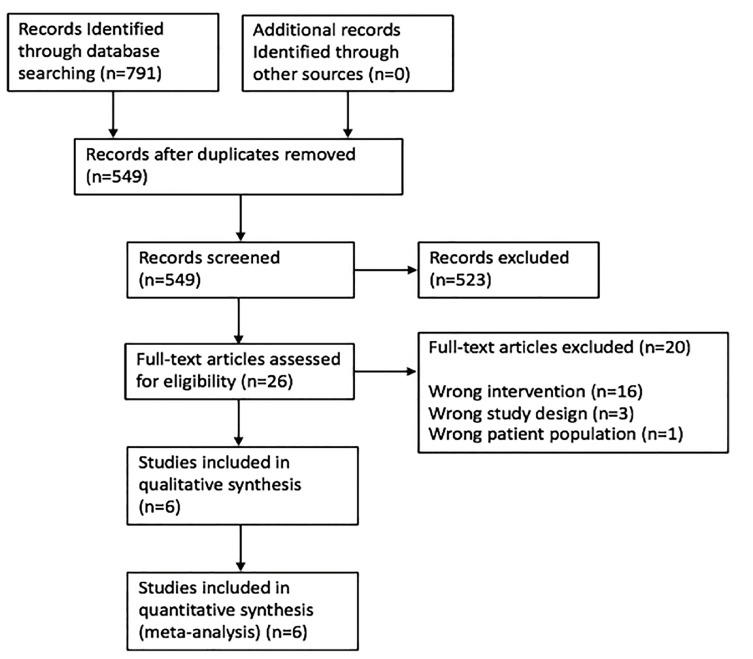
Preferred Reporting Items for Systematic Reviews and Meta-analyses (PRISMA) flow diagram. No additional articles were identified through bibliographic review.

We identified a total of 791 studies as follows: PubMed yielded 192; Embase 345; CINAHL 48; LILACS four; Google Scholar 200; the Cochrane Central Register of Controlled Trials two; and the Cochrane Database of Systematic Reviews yielded zero. After removal of duplicates, 549 abstracts were reviewed with 26 reviewed as full-text articles or conference abstracts (Figure 1).

Six studies comprising 1303 children were selected for the final analysis with a total of 155 cases (11.9%) of intussusception (Table 1).

Three studies were journal publications^16^,^18^,^19^ and three were meeting abstracts.^20^–^22^ Studies were conducted from 2010–2017 with the number of children in each study ranging from 44–775. Five studies were conducted in the United States^18^–^22^ and one was performed in Taiwan.^16^ All studies were performed in pediatric EDs. Three studies were retrospective,^16^,^19^,^21^ while three were prospective.^18^,^20^,^22^ The average age of patients ranged from 12.3 months to 6 years, with studies reporting male gender ranging from 59–68%. In five studies, sonographers were pediatric emergency physicians who had various levels of ultrasound training,^18^–^22^ with some having received relatively brief training on ultrasound while others had performed over 100 POCUS scans. In one study, the pediatric emergency physician performing POCUS was also a board-certified pediatric gastroenterologist.^16^ A linear transducer was used in three studies. The transducer type was not described in three studies. The reference standard varied between the six studies. Three studies^18^,^20^,^22^ used radiology-performed ultrasound as their gold standard, one study^19^ used radiology study (either computed tomography, ultrasound or barium enema), and another study^16^ used final diagnoses from the ED chart as well as chart review for admitted patients to the wards or return visits. Tryglidas et al^21^ used either radiology over-read of POCUS images or radiology-performed ultrasound as the reference standard.

**Figure 2:**
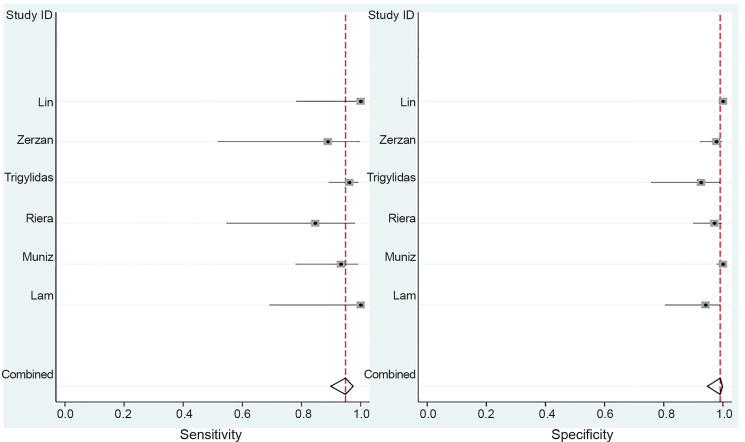
Forest plot with all included studies.

**Figure 3:**
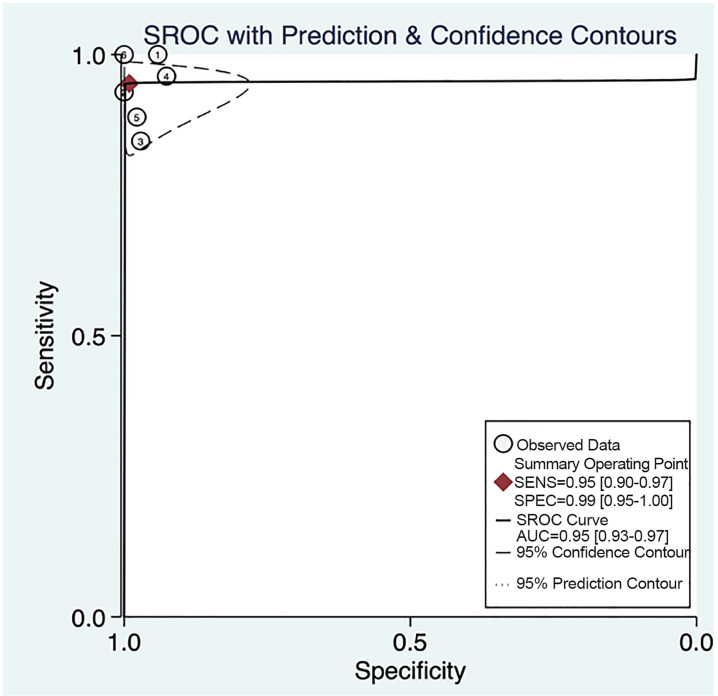
Summary receiver operating characteristic (SROC) curve with all studies. 1=Lam, 2=Muniz, 3=Riera, 4=Trigylidas, 5=Zerzan, 6=Lin. *SENS*, sensitivity; *SPEC*, specificity; *AUC*, area under the curve.

Overall POCUS was 94.9% sensitive (95% CI, 89.9% to 97.5%) and 99.1% specific (95% CI, 94.7%–99.8%) with a LR+ of 105 (95 % CI, 18–625) and a LR− of 0.05 (95% CI, 0.03–0.10) (Table 2, Figure 2). The area under the sROC curve was 0.95 (95% CI, 0.93 – 0.97), suggesting excellent diagnostic accuracy (Figure 3).

We also evaluated the data for PEM-only trained physicians, by excluding Lin et al^16^ (Table 2, Appendix Figure 1), given that the pediatric emergency physician in the study was also a board-certified pediatric gastroenterologist, and found similar sensitivity and specificity: 94.2% sensitive (88.5% to 97.2%) and 97.8% specific (94.1%–99.2%) with a LR+ of 43 (16–117) and a LR− of 0.06 (0.03–0.12) and area under the ROC curve of 0.97.

The study by Lin et al^16^ was at high risk for bias (Table 3). In terms of patient selection, this study included all patients with acute abdominal pain rather than those just with suspected intussusception. Out of 775 patients only 15, all under the age of three, were positive for intussusception, and it is unclear in how many children intussusception was suspected clinically. There were also applicability concerns for the index test, as the person who performed POCUS was board-certified in pediatric gastroenterology. Moreover, the diagnostic accuracy data included was for multiple different diagnoses including appendicitis, gastrointestinal infection, renal disease, gynecologic disease, gastrointestinal anomalies, extra-abdominal disease, and nonspecific abdominal pain, as well as for intussusception. Finally, for patients with negative POCUS, not all had received a follow-up radiology study and final diagnosis relied upon ED chart review, hospital course and possible revisits, which led to unclear bias in the reference standard. For these reasons, we also report pooled results after excluding this study (Table 2, Appendix Figure 1).

Additional sensitivity analysis of only prospective studies showed slightly lower sensitivity and similar specificity: 90.4% sensitive (79.0–96.8%) and 98.8% specific (96.9–99.7%) with a LR+ of 74 (28–197) and a LRv of 0.10 (0.04–0.22), and, sensitivity analysis of journal publications only, excluding abstracts, showed similar results to pooled data: 94.7% sensitive (82.3–99.4%) and 99.5% specific (98.8–99.9%) with a LR+ of 204 (77–545) and a LR− of 0.05 (0.01–0.20).

## DISCUSSION

This systematic review and meta-analysis demonstrates that POCUS for intussusception by pediatric emergency physicians is both highly sensitive and specific with accuracy similar to that of prior studies of radiology ultrasound for the diagnosis of intussusception.^23^ POCUS has the potential to reduce the time to treatment and overall length of stay in the ED. In fact, one study found that the institution of a POCUS protocol for intussusception reduced length of stay by over 200 minutes and shortened the door-to-reduction time by 26 minutes.^24^

A recent systematic review and meta-analysis by Tsou et al^23^ evaluated combined radiologic ultrasound and POCUS, demonstrating similar sensitivity and specificity to our study. However, our study differs in that we excluded radiology ultrasound and focused specifically on POCUS for intussusception. Additionally, the prior review included several studies with significant limitations, including one study^25^ that reported diagnostic accuracy data for patients who did not necessarily receive an ultrasound. In this retrospective study, patients were divided into two groups, one that was treated by pediatric EPs trained in POCUS for intussusception and one that was treated by pediatric EPs without this training. However, not all patients in the POCUS-trained group actually received a POCUS. The overall sensitivity for the group is reported, but not for the POCUS itself. The authors do report combined sensitivity and positive predictive value for POCUS by pediatric EPs and gastroenterology-performed ultrasound (considered the standard ultrasound in this study), but do not specifically assess the sensitivity and specificity of POCUS by pediatric EPs in isolation. We chose not to include this study for those reasons. Furthermore, they included the study by Lin et al^16^ that had a high risk of bias in the patient selection as well as applicability concerns for the index test and that was likely subject to an extreme form of incorporation bias.^26^ Given these concerns, we performed a sensitivity analysis excluding this study and report these results as well.

**Figure 5:**
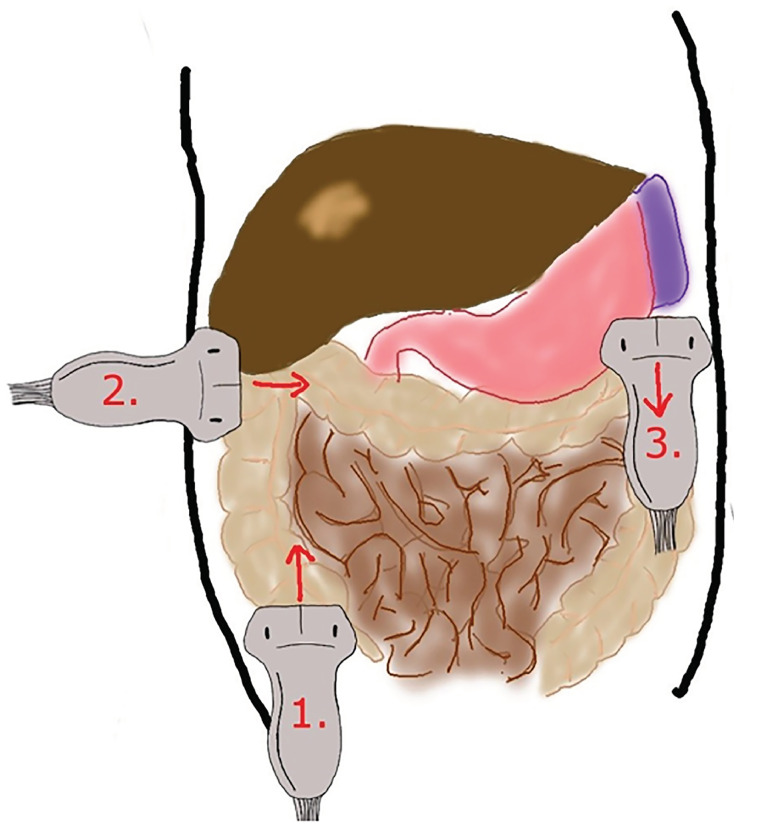
Intussusception scanning protocol. Initially published online on ALIEMU.com and permission to use given by author MLM.

When performing POCUS for intussusception, there is not currently a single preferred technique, although multiple have been described.^18^,^27^ These varying techniques can also affect the diagnostic accuracy of a test. We include a protocol (Figure 5) that was developed with POCUS experts and pediatric radiology at our institution.

Begin in the right lower quadrant, using a high-frequency linear probe with the probe marker to the patient’s right side. First, identify the psoas muscle and right iliac vessels as anatomical landmarks. Next, look for the transition from small bowel to large bowel and the ileocecal valve. Perform graded compression, with slow, steady pressure to displace bowel gas. Follow the colon from the right lower quadrant to right upper quadrant until the liver and gallbladder are identified. Rotate the probe marker to patient’s head and scan entire length of transverse colon. Rotate the probe marker back to patient’s right and scan entire length of descending colon, making sure to scan all four quadrants and to rescan any possible lesions.

Typically, an ileocolic intussusception appears as a “target sign” lesion, with one part of bowel (intussusceptum) telescoping into another part of bowel (intussuscipiens). In the transverse axis, the outer wall is thickened and hypoechoic. In the longitudinal axis, a “pseudokidney” sign has been described from the hyperechoic intussusceptum telescoping into the hypoechoic intussuscipiens. Other typical findings of ileocolic intussusception include lymph nodes in mesenteric fat noted in the intussusceptum.^3^

Based on our findings, POCUS could be considered for early diagnosis of intussusception. However, it is important to consider several limitations of POCUS for intussusception. These include operator dependence and the need for sufficient training. Future studies should establish the ideal training protocol and necessary number of POCUS exams for skill maintenance.

## LIMITATIONS

This study has several limitations that are important to consider. First, most studies did not state their specific scanning protocol, so it is unclear whether their specific protocols may have differed. Ultrasound, in general, is user-dependent and can vary based on training, skill, and frequency of practice. In our included studies, there was significant heterogeneity in who performed the POCUS, with some studies having experienced sonographers and others having physicians who had received short trainings. However, we believe this risk is low as prior studies have shown that ultrasound for intussusception can be learned by junior trainees^6^ and do not necessarily have to be performed by experts. Future studies should use standardized scanning protocols to limit variation and assess the test characteristics of physicians using these protocols.

Half of the included studies were abstracts rather than journal articles, which can limit ability to analyze sources of bias. However, when a separate sensitivity analysis was performed on journal articles only, we found similar results for diagnostic accuracy.

Additionally, half of the studies included were retrospective, which can bias the results. To help control for bias from retrospective studies, we also performed a sensitivity analysis without these studies, and the diagnostic accuracy data without retrospective studies showed slightly worse sensitivity, with larger CIs, but similar specificity. This change in sensitivity could be due to bias. The prevalence of intussusception varied among the included studies. The two studies, Lam et al^19^ and Trygylidas et al,^21^ with the highest prevalence of intussusception were both retrospective. This may suggest partial verification bias in the retrospective studies.^26^ One exception to this is Lin et al,^16^ which was retrospective, but had a low prevalence of intussusception (2%). However, this study included patients who may not have been suspected to have intussusception initially. Typically, a high prevalence of disease suggests partial verification bias where patients with positive index tests are more likely to get the reference standard test and patients with negative index tests are excluded from the study, meaning that true negatives are excluded (biasing specificity down) and false negatives are excluded (biasing sensitivity up). And indeed, both Lam et al^19^ and Trigylidas et al^21^ had relatively high sensitivities and low specificities.

The variation in prevalence of disease also suggests risk of selection bias, where included patients may have been selected who were more or less likely to have intussusception than the typical population where POCUS would be used, which also limits the generalizability of the results. The prospective studies included used convenience sampling based on when a physician trained in POCUS for intussusception was available. This may also limit the generalizability of this data. Also, there was moderate statistical heterogeneity between studies, which may also limit the generalizability of the data.

Larger, prospective studies, controlling for patient selection and physician training, are still needed for the accuracy of POCUS for intussusception. There was no data on patient outcomes or cost of care, and further trials are needed to determine the influence of POCUS on these factors. Finally, it is possible that some studies may have been missed with this search strategy. However, we used an extensive search strategy with the assistance of an experienced medical librarian, so we believe the risk of this is low.

## CONCLUSION

POCUS performed by emergency physicians is a highly sensitive and specific test for diagnosis of intussusception in children and has potential to be used as a screening tool. However, additional larger, prospective studies limiting bias are needed to assess the accuracy of POCUS for physicians of various training levels, using standardized protocols, and evaluating how use of POCUS for intussusception correlates with clinical outcomes.

## Supplementary Information



## Figures and Tables

**Figure 4:**
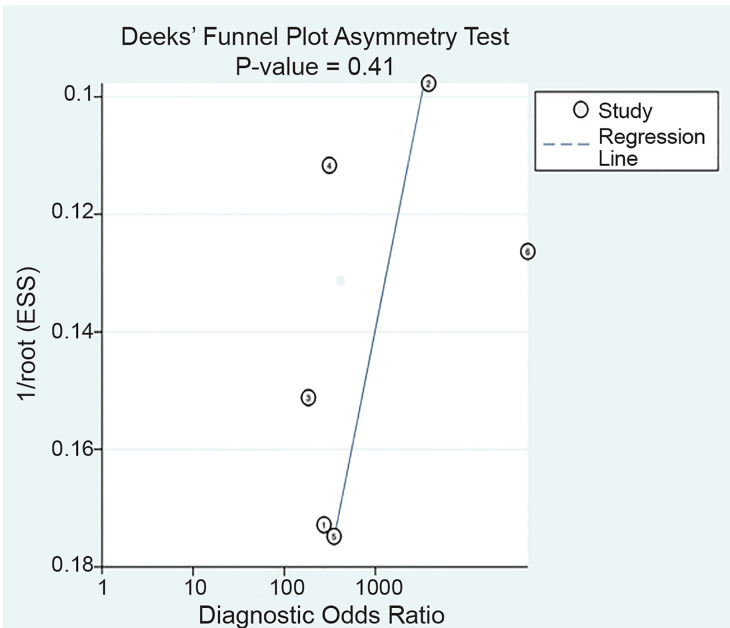
Funnel plot with all included studies. 1=Lam, 2=Muniz, 3=Riera, 4=Trigylidas, 5=Zerzan, 6=Lin. *ESS*, effective sample size.

**Table 1:** Characteristics of studies that assessed the accuracy of point-of-care ultrasound for diagnosis of intussuseption in children.

First author	Year	Study design	n	Country	Study location	Intussusception Rate	Average age	Male n (%)	Ultrasonographer training level	Ultrasound training protocol	Gold standard
Lam	2014	Retrospective	44	USA	PED	10 (23%)	31 months (mean)	30 (68%)	PEM Physicians	Minimum 1 hour of training on POCUS for ileocolic intussusception	Radiology study (CT, US or barium enema)
Muniz	2010	Prospective	198	USA	PED	30 (15.1%)	12.3 months	N/A	PEM physicians	N/A	Radiology US
Riera	2012	Prospective	82	USA	PED	13 (16%)	25 months (median)	48 (59%)	PEM attendings and fellows	1 month of clinical instruction POCUS, 100–150 adult exams, 1 hour focused bowel US training session by a pediatric radiologist	Radiology US
Trigylidas	2017	Retrospective	105	USA	PED	78 (74%)	22 months (mean)	67 (64.4%)	PEM Physicians	Trained in standard POCUS and underwent brief additional education in identification of ileocolic intussusception	Pediatric radiology direct overread of POCUS scan or radiology department ultrasound
Zerzan	2012	Prospective	99	USA	PED	9 (9%)	N/A	N/A	PEM attendings and fellows	PI gave brief in-service consisting of a didactic and hands-on ultrasound training session for all PEM attendings and fellows	Radiology US
Lin	2013	Retrospective	775	Taiwan	PED	15 (2%)	6 years (mean)	478 (62%)	PEM doctor also was Pediatric gastreoenterologist	Exams done by board certified pediatric GI physician	Chart review

*PED*, pediatric emergency department; *PEM*, pediatric emergency medicine; *POCUS*, point-of-care ultrasound; *PI*, principle investigator; *GI*, gastroenterology; *CT*, computed tomography; *US*, ultrasound; *N/A*, not available.

**Table 2:** Diagnostic accuracy data from included studies and pooled results.

Study	Sensitivity (95% CI)	Specificity (95% CI)	LR+ (95% CI)	LR− (95% CI)
Lam	100.0% (69.2%–100.0%)	94.1% (80.3%–99.3%)	17 (4–65)	
Muniz	93.3% (77.9%–99.2%)	100.0% (97.8%–100.0%)		0.07 (0.02–0.25)
Riera	84.6% (54.6%–98.1%)	97.1% (89.9%–99.6%)	29 (7–117)	0.16 (0.04–0.57)
Trigylidas	96.2% (89.2%–99.2%)	92.6% (75.7%–99.1%)	13 (3–49)	0.04 (0.01–0.13)
Zerzan	88.9% (51.8%–99.7%)	97.8% (92.2%–99.7%)	40 (10–161)	0.11 (0.02–0.72)
Lin	100.0% (78.2%–100.0%)	100.0% (99.5%–100.0%)		
Pooled-ALL	94.9% (89.9%–97.5%)	99.1% (94.7%–99.8%)	105 (18–624)	0.05 (0.03–0.10)
PEM-trained only	94.2% (88.5%–97.2%)	97.8% (94.1%–99.2%)	43 (16–117)	0.06 (0.03–0.12)

*PEM*, pediatric emergency medicine; *CI*, confidence interval; *LR+*, positive likelihood ratio; *LR*−, negative likelihood ratio.

**Table 3:** Quality Assessment of Diagnostic Accuracy Studies-2 (QUADAS-2) for included studies.

First author	Risk of bias					Applicability Concerns		
	
	Year	Patient selection	Index test	Reference standard	Flow and timing	Patient selection	Index test	Reference standard
	
Lam	2014	U	L	L	L	L	L	L
Muniz	2010	U	L	L	U	L	L	L
Riera	2012	U	L	L	L	L	L	L
Trigylidas	2017	U	L	U	U	U	L	L
Zerzan	2012	U	L	L	L	L	L	L
Lin	2013	H	U	U	U	H	H	U

*L*, low; *H*, high; *U*, unclear.
