# Supplementary material for: Diagnostic Accuracy of Point-of-Care Ultrasound for Intussusception in Children Presenting to the Emergency Department: A Systematic Review and Meta-analysis
Source: West J Emerg Med. 2020 Jul 2;21(4):1008–16. doi: 10.5811/westjem.2020.4.46241 (PMC7390574; doi:10.5811/westjem.2020.4.46241)
Supplement: Supplementary file 1 [file wjem-21-1008-s001.docx]

**Appendix**


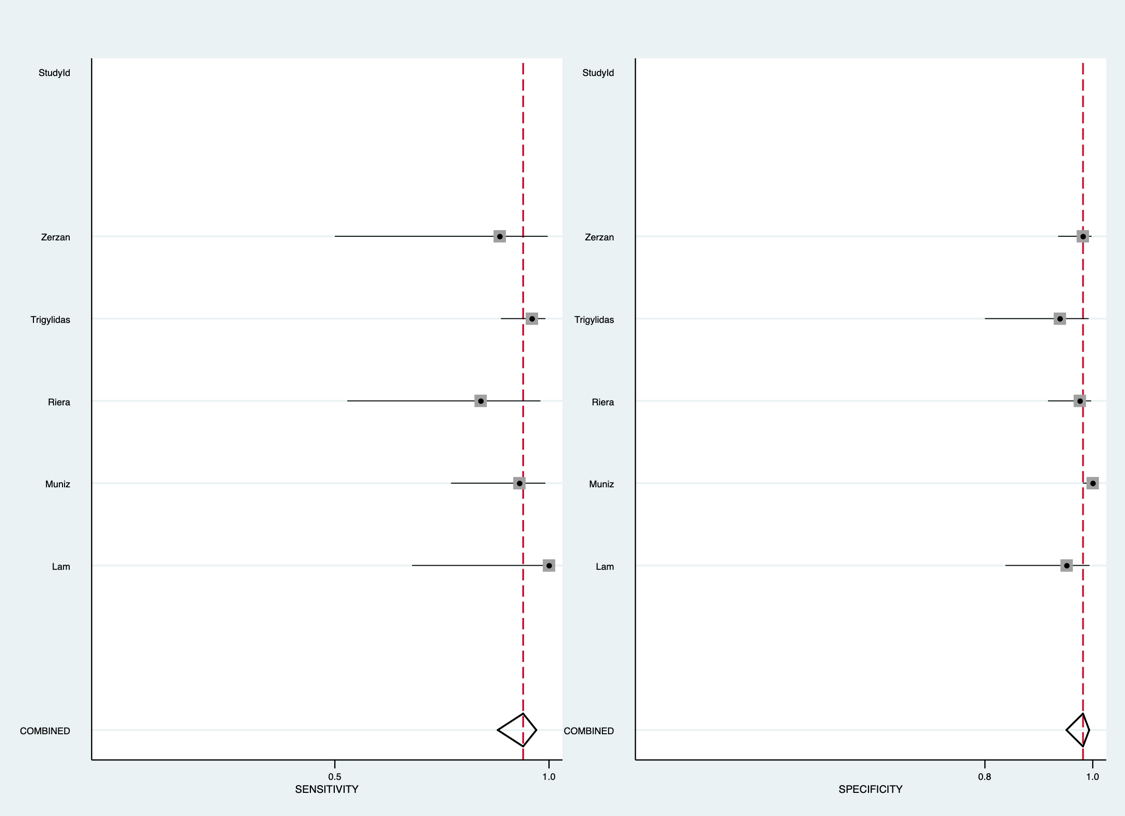


**Figure 1.** Forest Plot with PEM-trained Only Studies.
*PEM,* pediatric emergency medicine.

**Search Terms**

**Pubmed:**

"Intussusception"[Mesh] OR Intussusception) AND (ultraso* OR sonograp* OR "Ultrasonography"[Mesh]) AND (emergency OR bedside OR point-of-care OR "point of care")

**Embase:**

('intussusception'/exp OR intussusception) AND ultraso* AND (‘emergency’ OR “point of care” OR ‘point-of-care’ OR ‘bedside’)

**CINAHL**

(MH "Ultrasonography" OR “ultrasound” OR "sonograph*") AND (MH "intussusception") AND (‘emergency’ OR “point of care” OR ‘point-of-care’ OR ‘bedside’)

**LILACS**

(Ultrasonography Or ultrasound) AND intussusception AND (‘emergency’ OR “point of care” OR ‘point-of-care’ OR ‘bedside’)

**Google scholar (top 200 searches included)**

Intussusception AND (ultrasound OR sonography OR sonographer OR ultrasonography) AND (‘emergency’ OR “point of care” OR ‘point-of-care’ OR ‘bedside’)

**Cochrane Database of clinical trial and Cochrane Database of clinical reviews**

(intussusception):ti,ab,kw AND (ultrasound):ti,ab,kw AND ('emergency' OR 'point-of-care' OR 'bedside'):ti,ab,kw
